# Smoking Initiation, Tobacco Product Use, and Secondhand Smoke Exposure Among General Population and Sexual Minority Youth, Missouri, 2011–2012

**DOI:** 10.5888/pcd11.140037

**Published:** 2014-07-03

**Authors:** Jenna N. Jordan, Jane A. McElroy, Kevin D. Everett

**Affiliations:** Author Affiliations: Jane A. McElroy, Kevin D. Everett, University of Missouri, Department of Family and Community Medicine, Columbia, Missouri.

## Abstract

**Introduction:**

Research indicates disparities in risky health behaviors between heterosexual and sexual minority (referred to as LGBQ; also known as lesbian, gay, bisexual, queer, and questioning) youth. Limited data are available for tobacco-use–related behaviors beyond smoking status. We compared data on tobacco age of initiation, product use, and secondhand smoke exposure between general population and LGBQ youth.

**Methods:**

Data for general population youth were from the statewide, representative 2011 Missouri Youth Tobacco Survey, and data for LGBQ youth were from the 2012 Out, Proud and Healthy survey (collected at Missouri Pride Festivals). Age-adjusted Cochran-Mantel-Haenszel tests were used to examine differences between general population (N = 1,547) and LGBQ (N = 410) youth, aged 14 to 18 years. Logistic regression models identified variables associated with current smoking.

**Results:**

The 2 groups differed significantly on many tobacco-use–related factors. General population youth initiated smoking at a younger age, and LGBQ youth did not catch up in smoking initiation until age 15 or 16. LGBQ youth (41.0%) soon surpassed general population youth (11.2%) in initiation and proportion of current smokers. LGBQ youth were more likely to use cigars/cigarillos, be poly-tobacco users, and be exposed to secondhand smoke (SHS) in a vehicle (for never smokers). Older age (odds ratio [OR] = 1.39, 95% confidence interval [95% CI] = 1.18–1.62), female sex (OR = 1.64, 95% CI = 1.13–2.37), LGBQ identity (OR = 3.86, 95% CI = 2.50–5.94), other tobacco product use (OR = 8.67, 95% CI = 6.01–12.51), and SHS exposure in a vehicle (OR = 5.97, 95% CI = 3.83–9.31) all significantly increased the odds of being a current smoker.

**Conclusion:**

This study highlights a need for the collection of data on sexual orientation on youth tobacco surveys to address health disparities among LGBQ youth.

## Introduction

A growing body of research shows a higher proportion of smoking among sexual minority (referred to as LGBQ; also known as lesbian, gay, bisexual, queer, and questioning) than among heterosexual youth (1–5), and this disparity persists in adulthood (6–8). These disparities exist regardless of whether LGBQ status is defined by identity, attraction, or sexual behavior (6). Similarly, differences in age of smoking initiation were found between LGBQ and heterosexual youth in a longitudinal cohort study of adolescents, though data are sparse (1). Other sources of nicotine used by youth include chew, snus, cigars, and cigarillos. Research on a representative sample of US high school youth examined the concurrent use of multiple tobacco products; data indicated the prevalence of poly-tobacco use to be 21.7% among sexual minority youth compared with only 12.1% among heterosexual youth (9).

During the past 2 decades, exposure to secondhand smoke (SHS) has been linked to adverse health outcomes in both adults and children (10). Current research that is nationally representative of US youth has demonstrated that approximately 40% of middle- and high-school–age students are exposed to SHS in the home (11). Less is known about youth SHS exposure in vehicles; for example, a study using the nationally representative National Youth Tobacco Survey reported that one-fifth of nonsmoking youth are exposed to SHS in cars (12). No data on SHS exposure in the home or vehicle by LGBQ status were identified.

Because studies of risky behaviors and tobacco use among youth often collect data on a range of social and health issues, the information on tobacco use is limited and rarely includes measures of poly-tobacco use, SHS exposure, or support for smoke-free policies. The objective of this study was to 1) compare use of tobacco products, poly-tobacco use, and SHS exposure between Missouri general population and LGBQ youth; and 2) explore the relationship between smoking initiation, age, and LGBQ status among Missouri youth.

## Methods

The Missouri Youth Tobacco Survey (MYTS) has been conducted by the Missouri Department of Health and Senior Services with the Centers for Disease Control and Prevention (CDC) every other year since 2003. A cluster probability sampling plan was used and included all regular and charter public schools in Missouri. Schools were randomly selected to participate on the basis of a probability proportional to the school enrollment size. In the second stage of random sampling, classrooms in these schools were randomly selected. The overall participation proportion for survey completion was 72.1% (13).

Attendees at 6 Pride Festivals in Missouri (Kansas City, Columbia, Springfield, Joplin, St. Louis, and Black Pride St. Louis) during the summer of 2012 were asked to complete an anonymous 28-item paper survey known as the Out, Proud and Healthy (OPAH) survey. The Behavioral Risk Factor Surveillance System survey (14) was used as a guide for developing questions about tobacco use histories and SHS exposure. Basic demographic information was also collected. Compensation was not provided, but participants were offered bubbles, stickers, and ice water. The survey took approximately 10 minutes to complete. The eligibility criterion was being able to read English. The University of Missouri Health Sciences institutional review board approved the study for participants of all ages.

The MYTS did not collect data on sexual orientation. Individuals in the OPAH study were classified as sexual minorities on the basis of their response to the question “Do you consider yourself to be (lesbian, gay, bisexual, straight/heterosexual, other/please specify, or don’t know/not sure).” LGBQ status was defined as giving a response other than “straight/heterosexual.” The MYTS and OPAH surveys differed somewhat on their operationalization of tobacco-related variables, though the measures were comparable (Appendix).

Descriptive statistics and Cochran-Mantel-Haenszel tests were used to examine age-adjusted differences between the general population and LGBQ respondents on variables related to smoking initiation, tobacco product use, and SHS exposure. Only data on high school–aged youth (aged 14–18 years) were analyzed. Logistic regression models identified variables associated with current smoking. Additional regression analyses were conducted to identify variables associated with current smoking for youth aged 14 to 17 years and aged 18 years. Analyses were conducted using SAS 9.3 statistical software (SAS Institute, Inc, Cary, North Carolina).

## Results

Of the 5,243 OPAH surveys collected, participants who were missing data on age or LGBQ status (N = 228), aged 13 years or younger (N = 24), aged 19 years or older (N = 4,389), heterosexual youth (N = 166), youth completing the survey online (N = 5), and youth identifying as transgender or transsexual (N = 21) were excluded from analyses, bringing the final data set of LGBQ youth to 410 participants. The LGBQ sample included 113 lesbian (28%), 96 gay (23%), 143 bisexual (35%), and 58 other LGBQ/don’t know/not sure (14%) youth. The LGBQ and general population youth had a similar racial composition; most identified as white (LGBQ, 76.6%; general population, 79.5%). The 2 groups differed significantly by age (LGBQ mean age, 16.9 years; general population age, 16.2 years), Hispanic ethnicity (LGBQ, 6.7%; general population, 2.3%), and sex (LGBQ, 30.5% male; general population, 54.5% male) (Table 1).

**Table 1:** Cochran-Mantel-Haenszel Age-Controlled Comparisons of LGBQ and Missouri Youth on Demographics, Tobacco Use, Secondhand Smoke Exposure, and Attitudes About Smoking, Missouri Youth Tobacco Survey and Out, Proud and Healthy Survey, Missouri, 2011–2012^a^

Characteristic/Behavior/Attitude	LGBQ (N = 410)	General Population (N = 1,547)
**Mean age, y (standard deviation)^b^**	16.9 (1.1)	16.2 (1.1)
**Sex^c^**		
Male^b^	30.5	54.5
Female^b^	69.5	45.5
**Race^c^**		
Black	19.0	16.7
Asian	1.1	1.6
White	76.6	79.5
American Indian	3.4	1.6
Native Hawaiian	0	0.6
**Ethnicity^c^**		
Hispanic^b^	6.7	2.3
**Have you smoked at least 100 cigarettes?^c^**		
Yes^b^	31.8	12.8
**Smoking status^c^**		
Never^b^	55.5	87.3
Former	3.5	1.6
Current^b^	41.0	11.2
**Current smoker at age, y**		
14	0.8	2.5
15	3.1	13.6
16^b^	19.5	29.3
17^b^	28.9	30.3
18^b^	47.7	24.2
**Current smoker^c^**		
Every day^b^	52.6	33.7
Some days^d^	47.4	66.3
**Age of smoking initiation (current smokers), y^c^**		
8 or younger	1.4	6.0
9 or 10	3.5	7.5
11 or 12	17.5	18.3
13 or 14	30.1	31.5
15 or 16	32.9	30.1
17 or older^d^	14.7	6.6
**On the days you smoke, how many cigarettes do you smoke (current smokers)?^c^**		
5 or fewer	56.1	55.0
6–10	23.8	29.2
11–20	14.0	10.5
≥21	6.1	5.3
**Do you currently use chewing tobacco, snuff, or snus (male respondents only)?^c^**		
Not at all^d^	94.3	82.1
Every day^b^	0	7.3
Some days	5.7	10.6
**Do you currently use chewing tobacco, snuff, or snus (female respondents only)?^c^**		
Not at all^d^	97.8	98.0
Every day^d^	1.1	0.4
Some days	1.1	1.6
**Do you currently smoke cigars or cigarillos (male respondents only)?^c^**		
Not at all	73.1	82.9
Every day	1.7	2.0
Some days	25.2	15.1
**Do you currently smoke cigars or cigarillos (female respondents only)?^c^**		
Not at all^b^	70.4	92.0
Every day^b^	2.2	0.1
Some days^b^	27.4	7.8
**Poly-tobacco use (male respondents only)?^c^**		
Yes^b^	25.8	9.4
**Poly-tobacco use (female respondents only)?^c^**		
Yes^b^	19.8	4.4
**Support for smoke-free indoor workplace policies^c^**		
Yes^b^	54.0	75.3
**Which statement best describes the rules about smoking when you travel (never smokers)? Smoking is . . .^c^**		
Not allowed in any vehicle I travel in^b^	46.5	68.0
Allowed sometimes^b^	32.4	16.7
Always allowed	21.1	15.2
**Which statement best describes the rules about smoking when you travel (current smokers)? Smoking is . . .^c^**		
Not allowed in any vehicle I travel in	9.8	14.2
Allowed sometimes^d^	49.6	25.4
Always allowed^b^	40.7	60.4
**Which statement best describes the rules about smoking inside your home (never smokers)? Smoking is . . .^c^**		
Not allowed^b^	77.7	74.0
Sometimes allowed	10.8	11.5
Always allowed	11.5	14.5
**Which statement best describes the rules about smoking inside your home (current smokers)? Smoking is . . .^c^**		
Not allowed	76.3	41.1
Sometimes allowed	16.1	19.6
Always allowed^b^	7.6	39.3

Abbreviation: LGBQ, lesbian, gay, bisexual, queer, and questioning.

a Values expressed as percentages, unless otherwise indicated. Percentages may not sum to 100% due to rounding.

b *P* < .001

c Variable controlled for age.

d *P* < .01.

**Figure 1:**
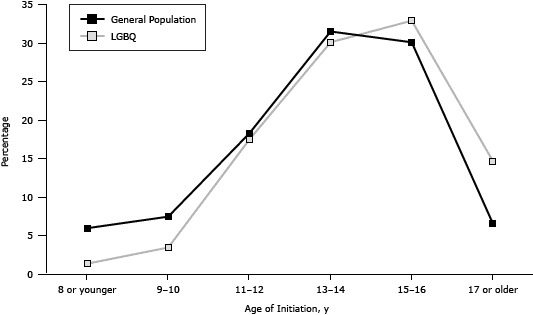
Age of smoking initiation (current smokers only) among LGBQ and general population youth, Missouri Youth Tobacco Survey and Out, Proud and Healthy Survey, Missouri, 2011–2012. Abbreviation: LGBQ, lesbian, gay, bisexual, queer and questioning. **Table:** 

**Figure 2:**
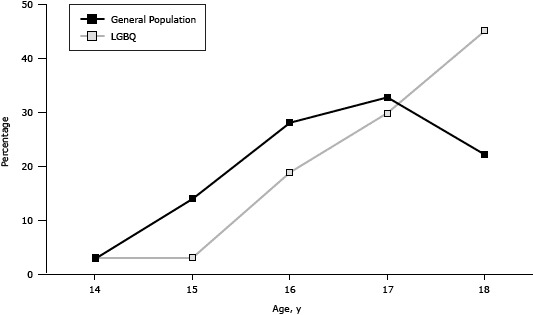
Percentage of current smokers, by age, among LGBQ and general population youth, Missouri Youth Tobacco Survey and Out, Proud and Healthy Survey, Missouri, 2011–2012. Abbreviation: LGBQ, lesbian, gay, bisexual, queer and questioning. **Table:** 

No differences in the proportion of current smokers were found by sex in LGBQ youth (male participants, 38.8%; female participants, 42.2%) or general population youth (male participants, 11.9%; female participants, 10.5%). The groups differed significantly, however, on many smoking status variables (Table 1). Overall, LGBQ youth were significantly more likely to have smoked at least 100 cigarettes in their lives. However, when examined by age, general population youth aged 14 to 17 were more likely than their LGBQ counterparts to have smoked at least 100 cigarettes. The percentage of youth initiating smoking at each age showed a similar pattern for LGBQ and general population youth, with a slight delay in initiation for LGBQ youth (Figure 1). At age 14, the percentage of current smokers was similar for the 2 groups (LGBQ, 0.8%; general population, 2.5%). For ages 15, 16, and 17, the percentage of current smokers was higher among the general population youth. Then at age 18, the percentage of current smokers among LGBQ youth (47.7%) nearly doubled that of youth from the general population (24.2%) (Figure 2). The overall LGBQ sample had a 41.0% current smoking prevalence compared with 11.2% in the general population, and, among smokers, LGBQ youth had a higher prevalence of daily smoking (LGBQ, 52.6%; general population, 33.7%).

Age of Initiation, y	General Population, %	LGBQ, %
8 or younger	6.0	1.4
9 or 10	7.5	3.5
11 or 12	18.3	17.5
13 or14	31.5	30.1
15 or 16	30.1	32.9
17 or older	6.6	14.7

Age, y	General Population, %	LGBQ, %
14	2.9	3.1
15	14.0	3.1
16	28.1	18.9
17	32.8	29.9
18	22.2	45.1

**Figure 3:**
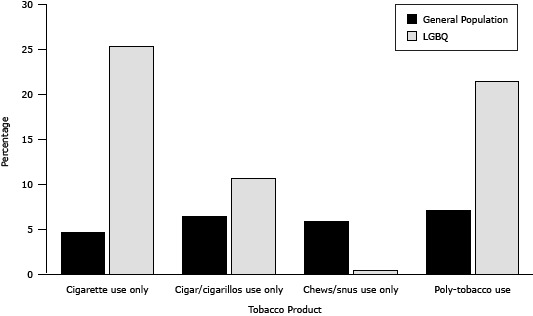
Types of Tobacco Use Among LGBQ and General Population Youth, Missouri Youth Tobacco Survey and Out, Proud and Healthy Survey, 2011–2012. Abbreviation: LGBQ, lesbian, gay, bisexual, queer and questioning. **Table:** 

In addition, LGBQ youth were significantly more likely than general population youth to smoke cigarettes (male and female participants), to smoke cigars/cigarillos (male and female participants), be poly-tobacco users (male and female participants (Table 1, Figure 3), be exposed to SHS in a vehicle (never smokers), and to report not supporting smoke-free indoor workplaces. Conversely, general population youth were significantly more likely to initiate smoking younger, use chewing tobacco (male participants), and be exposed to SHS in the home (never and current smokers). When SHS exposure in a vehicle for current smokers was examined dichotomously (never allowed vs sometimes allowed/always allowed), LGBQ and general population youth did not significantly differ.

Tobacco Product	General Population, %	LGBQ, %
Cigarette use only	4.7	25.3
Cigar/cigarillos use only	6.4	10.7
Chews/snus use only	5.9	0.5
Poly-tobacco use	7.1	21.5

Logistic regression analysis identified variables associated with current smoking. Age (OR = 1.39, 95% CI = 1.18–1.62), female sex (OR = 1.64, 95% CI = 1.13–2.37), LGBQ identity (OR = 3.86, 95% CI = 2.50–5.94), using a tobacco product other than cigarettes (OR = 8.67, 95% CI = 6.01–12.51), and allowing smoking in the vehicle (OR = 5.97, 95% CI = 3.83–9.31) all significantly increased the odds of being a current smoker. Conversely, black race (OR = 0.47, 95% CI = 0.26–0.84) and supporting smoke-free indoor workplace policies (0.48, 95% CI = 0.34–0.69) were protective against current smoking (Table 2). For 14- to 17-year-olds, the variables associated with current smoking remained the same as in the original model. However, for 18-year-olds, female sex was no longer significant in the model.

**Table 2:** Logistic Regression Model for Characteristics, Behaviors, and Attitudes Associated With Current Smoking in Missouri Youth, Missouri Youth Tobacco Survey and Out, Proud and Healthy Survey, Missouri, 2011–2012

Variable	Odds Ratio (95% Confidence Interval)	*P* Value
Older age (vs younger age)	1.39 (1.18–1.62)	<.001
Female sex (vs male sex)	1.64 (1.13–2.37)	.009
Black race (vs white race)	0.47 (0.26–0.84)	.01
LGBQ identity (vs general population)	3.86 (2.50–5.94)	<.001
Other tobacco use (vs no other tobacco use)	8.67 (6.01–12.51)	<.001
Support smoke-free indoor workplace policies (vs no support)	0.48 (0.34–0.69)	<.001
Smoking allowed in the vehicle (vs smoking not allowed in the vehicle)	5.97 (3.83–9.31)	<.001

Abbreviation: LGBQ, lesbian, gay, bisexual, queer, and questioning.

## Discussion

This study found LGBQ youth to be at an increased risk for smoking at least 100 lifetime cigarettes, current smoking, daily smoking, poly-tobacco use, cigar/cigarillo smoking, and exposure to SHS in a vehicle (for never smokers) compared with general population youth. However, the general population youth initiated smoking at a younger age, were more likely to use chew tobacco/snus (for male participants), and be exposed to SHS in the home. Consistent with findings from previous studies on smoking rates, our findings indicate that significantly more LGBQ youth than heterosexual youth are smoking by age 18. Our study extended findings on tobacco-use–related behaviors and exposures by showing a significant disparity in poly-tobacco use, other tobacco product use, and private-space SHS exposure for LGBQ youth compared with their general population counterparts.

The concurrent use of multiple tobacco products is a public health concern, because it could lead to greater nicotine dependence, which is especially true for youth who may be experimenting with various tobacco products and increasing their nicotine exposure (9). Approximately 2.5 times more LGBQ male youth and 5 times more LGBQ female youth were poly-tobacco users compared with their general population counterparts. Excluding current cigarette smokers, LGBQ youth were more likely to smoke cigars/cigarillos, and general population youth were more likely to use chew/snus. As a result, the much higher proportion of poly-tobacco use seen in the LGBQ sample is driven by the high proportion of cigarette smoking. However, youth who use more than 1 tobacco product are more likely to engage in health risk behaviors that are linked to substantial rates of morbidity and mortality in youth (15), suggesting LGBQ youth are at increased risk for illness and death.

The differences in tobacco use by LGBQ status may be the result of the socially based stressors that LGBQ youth face such as stigma, discrimination, and a lack of acceptance from friends and family. In the stress and coping model of adolescent substance abuse, people who experience high levels of stress and a lack of healthy coping resources (ie, social support) may use tobacco products in an attempt to cope with stress (16). A study by Rosario and colleagues followed a group of LGB youth longitudinally for 1 year to determine whether the heightened risk of cigarette smoking in this population resulted from the belief among LGB youth that smoking would serve as a coping strategy for the greater stress they experienced as a result of their sexual orientation (17). The results showed that smoking increased the negative association between stressors and psychological distress, although both friend and family support buffered this association (17). Despite perceptions among youth that smoking is an effective coping strategy for stress, smoking may have adverse consequences for distress (17), thus perpetuating the high smoking rates seen in this population.

LGBQ youth also experience high levels of rejection and less support from their parents and peers (18), which may contribute to the higher prevalence of tobacco use in this population. One convenience sample study found that parental rejection behaviors toward adolescent sexual orientation predicted negative health problems such as illegal drug use in young adulthood (19). In addition, at-school victimization has been shown to negatively influence LGBQ youths’ health risk behaviors (20) and increase suicidality (21).

Relationships among different facets of life are an important factor in tobacco use (22). Primary socialization theory emphasizes the relationships between individuals and their family, peers, and school faculty as the conduit through which information about norms and behaviors is conveyed (22). According to this theory, when the bonds between adolescents and others is strong and the influence is prosocial, youth are not as likely to engage in tobacco use; however, when these bonds are weak, the influence of peers increases along with the likelihood that these peers will be composed of youth who promote substance use behaviors (22). In this case, when nonsmoking youth are friends with smokers and have weak relationships with family and school faculty, they are more likely to become smokers themselves than are youth who do not associate with smokers (23). Although these theories have been applied to general population youth, they likely extend to LGBQ youth, who are also influenced by relationships with family, peers, and school faculty.

The lower proportion of LGBQ youth smoking before the age of 18 compared with general population youth may be explained by an instability of identity or self-concept (24), which does not stabilize until late adolescence (25). In this sense, identifying as LGBQ at a young age may create a sense of isolation that prevents these youth from learning smoking behaviors from peer groups. Ages 18 to 19 may be a time by which self-concept is established (26), when many LGBQ individuals disclose their sexual orientation (“come out”), leave their family home, and begin socializing with other LGBQ individuals and groups; as a result, they may begin smoking as a way of forging connections with new friends. In large part, youth view themselves in terms of their surrounding contexts when it comes to self-definition (27), and, by viewing LGBQ individuals as their peers, LGBQ youth may be modeling their tobacco use behaviors after a group that has been consistently shown to have a high prevalence (up to 50%) of tobacco use (7).

For LGBQ youth, tobacco product (single and combined) use and SHS exposure in cars for never smokers place this population at increased risk for tobacco-related illness (28). Despite fewer LGBQ youth being exposed to smoking in the home, a higher proportion of LGBQ youth smoke compared with general population youth, indicating that rules about smoking in the vehicle are more predictive of current smoking than rules about smoking in the home. This conclusion is also supported by the results of the regression analysis, which found that allowing smoking in the vehicle increased the odds of current smoking by 6 times, while allowing smoking in the home was not significant in the model. These findings also highlight the importance of peer influences on smoking. Although youth may have no control over the rules about smoking in their home, they may be able to determine rules about smoking in their own vehicles or friends’ vehicles where parental rules cannot always be enforced.

This study has several limitations. The operationalization of our OPAH tobacco-related measures sometimes differed from those of the MYTS, though both measure the same concepts. In addition, the MYTS reported findings for the general population, which would have included some LGBQ participants, introducing misclassification error. The small percentage of general population youth self-identifying as LGBQ is unlikely to substantially change our results, as LGBQ youth are at a higher risk for many tobacco-use and SHS variables, and, if any effect was seen, it would be a slight increase in general population risk. Despite this, the differences seen in tobacco and SHS variables remain significant between the groups. Finally, although this study has a large sample size of more than 400 self-identified LGBQ youth, the participants are a nonprobabilistic sample of participants, and the findings may not extend to all LGBQ Missouri youth.

The lack of nationally representative data on tobacco use in LGBQ youth results in few effective, evidence-based prevention, cessation, and counter-marketing campaigns for this group. In 2012, the American Legacy Foundation found that the only national data available on tobacco use and sexual orientation were from the National Adult Tobacco Survey, which omits youth (29). During 2011, only 21 sites (encompassing states, large school districts, territories, and tribal governments) in the United States asked students’ sexual identity and the sex of students’ sexual contacts on the Youth Risk Behavior Survey (YRBS) (30). The CDC encourages YRBS sites to add questions about same-sex sexual contact and sexual identity to their surveys to better understand the health risks among LGBQ youth and to develop appropriate prevention strategies (30). This study provides evidence for the inclusion of sexual orientation on youth tobacco and health surveys to address these disparities.

Future studies should further explore the reasons for delayed smoking initiation in LGBQ youth, and interventions should target LGBQ youth at a young age. Interventions that may be most effective for LGBQ youth are those that address the hazards of using tobacco products other than cigarettes (ie, cigars/cigarillos) and poly-tobacco use, as well as those that break the association between identifying as a member of the LGBQ community and smoking. It may also be beneficial to address issues unique to LGBQ females who often use tobacco products at equal or higher rates than LGBQ males. These interventions should include former smokers or nonsmokers from the LGBQ community and provide opportunities for LGBQ youth to socialize in smoke-free venues. At the individual level, LGBQ youth need to be provided with positive and healthy coping mechanisms to address the discrimination and stress they may experience. At the societal level, tobacco and SHS exposure disparities in the LGBQ community need to be treated as social justice and policy issues arising from widespread discrimination, victimization, and health care inequalities. Without identifying LGBQ status on health surveys and studies, health educators and practitioners cannot monitor trends in LGBQ youth tobacco product use, engage with this at-risk population, address the lack of LGBQ representation in mainstream tobacco control, or develop tailored prevention and cessation programs to reduce tobacco use in this population.

## Appendix. Differences in Measures of Missouri Youth Tobacco Survey and the Out, Proud and Healthy Survey, Missouri, 2011–2012

**Table:**

Variable	Missouri Youth Tobacco Survey	Out, Proud and Healthy Survey
**Smoking status**		
Never	About how many cigarettes have you smoked in your entire life? (response: none to 99 cigarettes)	Have you smoked at least 100 cigarettes in your entire life? (response: no)
Former	About how many cigarettes have you smoked in your entire life? (response: 100 or more cigarettes) *and* During the past 30 days, on how many days did you smoke? (response: 0 days)	Have you smoked at least 100 cigarettes in your entire life? (response: yes) *and* Do you NOW smoke cigarettes every day, some days or not at all? (response: not at all)
Current	About how many cigarettes have you smoked in your entire life? (response: 100 or more cigarettes) *and* During the past 30 days, on how many days did you smoke? (response: 1–30 days)	Have you smoked at least 100 cigarettes in your entire life? (response: yes) *and* Do you NOW smoke cigarettes every day, some days or not at all? (response: every day or some days)
**Current smoking**		
Not at all	During the past 30 days, on how many did you smoke? (response: 0 days)	Do you NOW smoke cigarettes every day, some days or not at all? (response: not at all)
Some days	During the past 30 days, on how many did you smoke? (response: 1–29 days)	Do you NOW smoke cigarettes every day, some days or not at all? (response: some days)
Every day	During the past 30 days, on how many did you smoke? (response: all 30 days)	Do you NOW smoke cigarettes every day, some days or not at all? (response: every day)
**Age of smoking initiation, y**		
8 or younger	How old were you when you smoked a whole cigarette for the first time? (response: 8 years or younger)	Age you started smoking? (response: 8 years or younger)
9 or 10	How old were you when you smoked a whole cigarette for the first time? (response: 9 or 10 years old)	Age you started smoking? (response: 9 or 10 years old)
11 or 12	How old were you when you smoked a whole cigarette for the first time? (response: 11 or 12 years old)	Age you started smoking? (response: 11 or 12 years old)
13 or 14	How old were you when you smoked a whole cigarette for the first time? (response: 13 or 14 years old)	Age you started smoking? (response: 13 or 14 years old)
15 or 16	How old were you when you smoked a whole cigarette for the first time? (response: 15 or 16 years old)	Age you started smoking? (response: 15 or 16 years old)
17 or older	How old were you when you smoked a whole cigarette for the first time? (response: 17-year-old or older)	Age you started smoking? (response: 17-year-old or older)
**Smoked 100 or more cigarettes**		
Yes	About how many cigarettes have you smoked in your entire life? (response: 100 or more cigarettes)	Have you smoked at least 100 cigarettes in your entire life? (response: yes)
No	About how many cigarettes have you smoked in your entire life? (response: none–99 cigarettes)	Have you smoked at least 100 cigarettes in your entire life? (response: no)
**Cigarettes per day**		
Do not smoke	During the past 30 days, on the days you smoked, how many cigarettes did you smoke per day? (response: I did not smoke cigarettes during the past 30 days)	On the days you smoke, how many cigarettes do you smoke? (response: I did not smoke at all)
5 or fewer	During the past 30 days, on the days you smoked, how many cigarettes did you smoke per day? (response: 5 or fewer)	On the days you smoke, how many cigarettes do you smoke? (response: 5 or fewer)
6–10	During the past 30 days, on the days you smoked, how many cigarettes did you smoke per day? (response: 6–10)	On the days you smoke, how many cigarettes do you smoke? (response: 6–10)
11–20	During the past 30 days, on the days you smoked, how many cigarettes did you smoke per day? (response: 11–20)	On the days you smoke, how many cigarettes do you smoke? (response: 11–20)
More than 20	During the past 30 days, on the days you smoked, how many cigarettes did you smoke per day? (response: more than 20)	On the days you smoke, how many cigarettes do you smoke? (response: more than 20)
**Chewing tobacco use**		
Not at all	During the past 30 days, on how many days did you use chewing tobacco, snuff, or dip? (response: 0 days)	Do you currently use chewing tobacco, snuff or snus every day, some days, or not at all? (response: not at all)
Some Days	During the past 30 days, on how many days did you use chewing tobacco, snuff, or dip? (response: 1–29 days)	Do you currently use chewing tobacco, snuff or snus every day, some days, or not at all? (response: some days)
Every day	During the past 30 days, on how many days did you use chewing tobacco, snuff, or dip? (response: all 30 days)	Do you currently use chewing tobacco, snuff or snus every day, some days, or not at all? (response: every day)
**Cigar use**		
Not at all	During the past 30 days, on how many days did you smoke cigars, cigarillos, or little cigars? (response: 0 days)	Do you now smoke cigars, water pipe, bidis, kreteks, or clove cigarettes every day, some days, or not at all? (response: not at all)
Some days	During the past 30 days, on how many days did you smoke cigars, cigarillos, or little cigars? (response: 1–29 days)	Do you now smoke cigars, water pipe, bidis, kreteks, or clove cigarettes every day, some days, or not at all? (response: some days)
Every day	During the past 30 days, on how many days did you smoke cigars, cigarillos, or little cigars? (response: all 30 days)	Do you now smoke cigars, water pipe, bidis, kreteks, or clove cigarettes every day, some days, or not at all? (response: every day)
**Poly-tobacco use**		
Yes	During the past 30 days, on how many did you smoke? (response: 1 to all 30 days) *and* During the past 30 days, on how many days did you use chewing tobacco, snuff, or dip? (response: 1 to all 30 days) *or* During the past 30 days, on how many days did you smoke cigars, cigarillos, or little cigars? (response: 1 to all 30 days)	Do you NOW smoke cigarettes every day, some days or not at all (response: every day or some days) *and* Do you currently use chewing tobacco, snuff or snus every day, some days, or not at all? (response: every day or some days) *or* Do you now smoke cigars, water pipe, bidis, kreteks, or clove cigarettes every day, some days, or not at all? (response: every day or some days)
**Exposure to secondhand smoke in the home**		
Allowed some times or places	Which of these best describes the rules about smoking inside the house where you live? Smoking is . . . (response: allowed only at some times or in some places)	Which statement best describes the rules about smoking inside your home? (response: allowed in some places or at some times)
Always allowed	Which of these best describes the rules about smoking inside the house where you live? Smoking is . . . (response: always allowed inside my home)	Which statement best describes the rules about smoking inside your home? (response: allowed in all places inside my home/there are no rules about smoking inside my home)
Never allowed	Which of these best describes the rules about smoking inside the house where you live? Smoking is… (response: never allowed inside my home)	Which statement best describes the rules about smoking inside your home? (response: not allowed anywhere inside my home)
**Exposure to secondhand smoke in the car**		
Allowed some times or places	Which of the following best describes the rules about smoking in the vehicle you drive or ride in the most? Smoking is… (response: sometimes allowed inside the vehicle)	Which statement best describes the rules about smoking when you travel? (response: not allowed in my vehicle but smoking can happen when I am traveling in another person’s vehicle/allowed sometimes, depending on the person driving or the situation)
Always allowed	Which of the following best describes the rules about smoking in the vehicle you drive or ride in the most? Smoking is… (response: always allowed inside the vehicle)	Which statement best describes the rules about smoking when you travel? (response: always allowed in my vehicle)
Never allowed	Which of the following best describes the rules about smoking in the vehicle you drive or ride in the most? Smoking is… (response: never allowed inside the vehicle)	Which statement best describes the rules about smoking when you travel? (response: not allowed in my vehicle in which I travel)
**Support for smoke-free workplaces**		
Yes	What do you think employers should do about smoking in indoor areas where people work? Employers should . . . (response: never allow smoking in places where people work)	Do you support smoke-free policies in all indoor workplaces, including bars and restaurants? (response: yes)
No	What do you think employers should do about smoking in indoor areas where people work? Employers should . . . (response: allow smoking only at some times or in some places/always allow smoking in places where people work)	Do you support smoke-free policies in all indoor workplaces, including bars and restaurants? (response: no)

## References

[1] Corliss HL , Wadler BM , Jun HJ , Rosario M , Wypij D , Frazier AL , Sexual-orientation disparities in cigarette smoking in a longitudinal cohort study of adolescents. Nicotine Tob Res 2013;15(1):213–22. 10.1093/ntr/nts114 22581940 PMC3524066

[2] Austin SB , Ziyadeh N , Fisher LB , Kahn JA , Colditz GA , Frazier AL . Sexual orientation and tobacco use in a cohort study of US adolescent girls and boys. Arch Pediatr Adolesc Med 2004;158(4):317–22. 10.1001/archpedi.158.4.317 15066869

[3] Easton A , Jackson K , Mowery P , Comeau D , Sell R . Adolescent same-sex and both-sex romantic attractions and relationships: implications for smoking. Am J Public Health 2008;98(3):462–7. 10.2105/AJPH.2006.097980 18235075 PMC2253579

[4] Brewster KL , Tillman KH . Sexual orientation and substance use among adolescents and young adults. Am J Public Health 2012;102(6):1168–76. 10.2105/AJPH.2011.300261 22021322 PMC3483938

[5] Ryan H , Wortley PM , Easton A , Pederson L , Greenwood G . Smoking among lesbians, gays, and bisexuals: a review of the literature. Am J Prev Med 2001;21(2):142–9. 10.1016/S0749-3797(01)00331-2 11457635

[6] Lee JG , Griffin GK , Melvin CL . Tobacco use among sexual minorities in the USA, 1987 to May 2007: a systematic review. Tob Control 2009;18(4):275–82. 10.1136/tc.2008.028241 19208668

[7] McElroy JA , Everett KD , Zaniletti I . An examination of smoking behavior and opinions about smoke-free environments in a large sample of sexual and gender minority community members. Nicotine Tob Res 2011;13(6):440–8. 10.1093/ntr/ntr021 21372088

[8] Rath JM , Villanti AC , Rubenstein RA , Vallone DM . Tobacco use by sexual identity among young adults in the United States. Nicotine Tob Res 2013;Online First. 10.1093/ntr/ntt062 23680918

[9] Rosario M , Corliss HL , Everett B , Reisner SL , Austin SB , Buchting FO , Sexual orientation disparities in cancer-related risk behaviors of tobacco, alcohol, sexual behaviors, and diet and physical activity: pooled youth risk behavior surveys. Am J Public Health 2014;104(2):245–54. 10.2105/AJPH.2013.301506 24328632 PMC3935697

[10] Sureda X , Fernandez E , Lopez MJ , Nebot M . Secondhand tobacco smoke exposure in open and semi-open settings: a systematic review. Environ Health Perspect 2013;121(7):766–73. 10.1289/ehp.1205806 23651671 PMC3701994

[11] Agaku IT , Vardavas CI . Disparities and trends in indoor exposure to secondhand smoke among US adolescents: 2000–2009. PLoS ONE 2013;8(12):e83058. 10.1371/journal.pone.0083058 24358249 PMC3866255

[12] King BA , Dube SR , Tynan MA . Secondhand smoke exposure in cars among middle and high school students — United States, 2000–2009. Pediatrics 2012;129(3):446–52. 10.1542/peds.2011-2307 22311992 PMC4583774

[13] Results from the 2011 Missouri Youth Tobacco Survey. Missouri Department of Health and Senior Services, Office of Epidemiology; 2011. http://health.mo.gov/living/wellness/tobacco/smokingandtobacco/pdf/2011YouthTobaccoSurvey.pdf. Updated December 2011. Accessed August 22, 2013.

[14] Behavioral risk factor surveillance system survey questionnaire. US Department of Health and Human Services, Centers for Disease Control and Prevention; 2008. http://www.cdc.gov/brfss/questionnaires/pdf-ques/2008brfss.pdf. Updated December 31, 2007. Accessed August 6, 2013.

[15] Everett SA , Malarcher AM , Sharp DJ , Husten CG , Giovino GA . Relationship between cigarette, smokeless tobacco, and cigar use, and other health risk behaviors among US high school students. J Sch Health 2000;70(6):234–40. 10.1111/j.1746-1561.2000.tb07424.x 10937370

[16] Wills TA , Filer M . Stress-coping model of adolescent substance use. In: Ollendick TH, Prinz RJ, editors. Advances in clinical child psychology, vol. 18. New York (NY): Plenum Press; 1996. p. 91–132.

[17] Rosario M , Schrimshaw EW , Hunter J . Cigarette smoking as a coping strategy: negative implications for subsequent psychological distress among lesbian, gay and bisexual youths. J Pediatr Psychol 2011;36(7):731–742. 10.1093/jpepsy/jsp141 20123704 PMC3146751

[18] Williams T , Connolly J , Pepler D , Craig W . Peer victimization, social support, and psychosocial adjustment of sexual minority adolescents. J Youth Adolesc 2005;34(5):471–82. 10.1007/s10964-005-7264-x

[19] Ryan C , Hueber D , Diaz RM , Sanchez J . Family rejection as a predictor of negative health outcomes in white and Latino lesbian, gay, and bisexual young adults. Pediatrics 2009;123(1):346–52. 10.1542/peds.2007-3524 19117902

[20] Bontempo DE , D’Augelli AR . Effects of at-school victimization and sexual orientation on lesbian, gay, or bisexual youths’ health risk behavior. J Adolesc Health 2002;30(5):364–74. 10.1016/S1054-139X(01)00415-3 11996785

[21] Russell ST , Joyner K . Adolescent sexual orientation and suicide risk: evidence from a national study. Am J Public Health 2001;91(8):1276–81. 10.2105/AJPH.91.8.1276 11499118 PMC1446760

[22] Oetting ER , Deffenbacher JL , Donnermeyer JF . Primary socialization theory. The role played by personal traits in the etiology of drug use and deviance. II. Subst Use Misuse 1998;33(6):1337–66. 10.3109/10826089809062220 9603274

[23] Urberg KA , Degirmencioglu SM , Pilgrim C . Close friend and group influence on adolescent cigarette smoking and alcohol use. Dev Psychol 1997;33(5):834–44. 10.1037/0012-1649.33.5.834 9300216

[24] Rosenberg M . Self-concept and psychological well-being in adolescence. In: Leahy RL, editor. The development of the self. Orlando (FL): Academic Press; 1985. p. 205–46.

[25] Boardman JD . Self-rated health among US adolescents. J Adolesc Health 2006;38(4):401–8. 10.1016/j.jadohealth.2005.01.006 16549301 PMC3157914

[26] Stryker S . Symbolic interactionism: a social structural version. Menlo Park (CA): Benjamin/Cummings; 1980.

[27] Damon W , Hart D . Self-understanding in childhood and adolescence. New York (NY): Cambridge University Press; 1988.

[28] Child and teen tobacco use. American Cancer Society; 2012. http://www.cancer.org/acs/groups/cid/documents/webcontent/002963-pdf.pdf. Updated November 8, 2012. Accessed August 21, 2013.

[29] Tobacco fact sheet: lesbian, gay, bisexual, and transgender (LGBT) communities and smoking. Washington (DC): American Legacy Foundation; 2012. http://www.legacyforhealth.org/content/download/593/7117/file/LGBT_Communities_and_Smoking_2012.pdf.pdf. Updated November 2012. Accessed August 19, 2013.

[30] LGBTQ youth programs-at-a-glance. Centers for Disease Control and Prevention; 2013. http://www.cdc.gov/lgbthealth/youth-programs.htm. Updated May 16, 2013. Accessed August 21, 2013.

